# Microarray Analysis of lncRNA and mRNA Expression Profiles in Patients with Neuromyelitis Optica

**DOI:** 10.1007/s12035-016-9754-0

**Published:** 2016-03-03

**Authors:** Jing Xu, Fang Zhang, Chao Gao, Xiaofeng Ma, Xiaolin Peng, Dexin Kong, Junwei Hao

**Affiliations:** 1 Department of Neurology, Tianjin Neurological Institute, Tianjin Medical University General Hospital, Tianjin, 300052 China; 2 Tianjin Key Laboratory on Technologies Enabling Development of Clinical Therapeutics and Diagnostics, School of Pharmacy, Tianjin Medical University, Tianjin, 300070 China

**Keywords:** Neuromyelitis optica, Long noncoding RNAs, Messenger RNAs, Microarray, Gene ontology, Pathway analysis

## Abstract

For the epigenetic characterization of neuromyelitis optica (NMO), we determined whether messenger RNAs (mRNAs) and long noncoding RNAs (lncRNAs) are expressed differentially in subjects with and without NMO. lncRNA and mRNA expression profiles of NMO patients and healthy controls were generated by using microarray analysis. For comparison, the differentially expressed mRNA functions were annotated by using gene ontology (GO) and pathway analyses. The microarray revealed that 1310 lncRNAs and 743 mRNAs differed in NMO patients from those in healthy controls. Pathway analysis then demonstrated that IL23-mediated signaling events, interferon gamma signaling, natural killer (NK)-κB signaling pathway, chemokine receptors that bind chemokines, GPCR ligand binding, and metabolic disorders of biological oxidation enzyme pathways play important roles in NMO. Several GO terms including cytokine stimulus, response to cytokine, immune response, cytokine-mediated signaling pathway, and response to chemical cytokine activity were enriched in gene lists, suggesting a potential correlation with NMO. Co-expression network analysis indicated that 183 lncRNAs and 458 mRNAs were included in the co-expression network. Our present study showed that these differentially expressed mRNAs and lncRNAs may play important roles in NMO and could provide basic information for new biomarkers or treatment targets to alleviate NMO.

**Electronic supplementary material:**

The online version of this article (doi:10.1007/s12035-016-9754-0) contains supplementary material, which is available to authorized users.

## Introduction

Neuromyelitis optica (NMO) is characterized by severe attacks of optic neuritis and/or longitudinally extensive transverse myelitis [1]. A significant proportion of NMO patients are seropositive for antibodies that target aquaporin-4 [2], the main water channel in the central nervous system (CNS), and specifically localize to astrocyte processes [3]. However, the epigenetic characteristics of this disease are not completely understood.

Long noncoding RNAs (lncRNAs) are typified by a length of transcription longer than 200 nucleotides that is not translated into proteins [4]. Increasing scientific interest in these factors stems from previous investigations showing that lncRNAs exert their regulatory effects on gene expression levels, involving epigenetic regulation, transcriptional regulation, and post-transcriptional regulation in the form of RNA [5]. IncRNAs also play important roles in modulating innate and adaptive immune responses and immune cell development [6]. Moreover, emerging evidence suggests the decisive participation of lncRNAs in such autoimmune diseases as systemic lupus erythematosus (SLE), rheumatoid arthritis (RA), type 1 diabetes mellitus (T1DM), and multiple sclerosis (MS) [7].

A comprehensive understanding of the epigenetic and molecular disorders of the disease is the key to early diagnosis, appropriate treatment, and better prognosis for patients with NMO. Therefore, the present study was initiated to use lncRNA microarray for the characterization of genome-wide lncRNA and messenger RNA (mRNA) expression profiles of NMO patients compared with healthy controls. Our goal was to establish the potential utility of lncRNAs as biomarkers of or treatment targets for NMO.

## Materials and Methods

### Patients and Sample Collection

For this study, we enrolled 16 patients who had been diagnosed with NMO according to the revised diagnostic criteria as proposed by Wingerchuk [8] in 2015 in Tianjin Medical University General Hospital between 2014 and 2015. These patients were within the peak timing of manifesting NMO and before treatment with glucocorticoid or intravenous immune globulin. We also recruited 16 age- and gender-matched healthy controls for the comparative study. The demographic and clinical features of all the patients and healthy controls are summarized in Table 1. Sera antiaquaporin 4 (AQP4) antibody was detected by the cell-based assay (CBA) as described in a previous study [9]. Informed consent was obtained at enrollment from all patients or legally acceptable surrogates. Peripheral blood anticoagulated with ethylene diamine tetraacetic acid (EDTA) was obtained from all NMO patients and healthy controls. Human peripheral blood mononuclear cells (PBMCs) were isolated with Ficoll-Hypaque gradients. The present study was approved by the ethics committee of Tianjin Medical University General Hospital.

**Table 1:** Baseline characteristics

	Control (*n* = 16)	NMO (*n* = 16)	*P* value
Gender, F/M	14/2	14/2	1.00
Age at onset, median (range) (years) Disease duration, median (range) (years) Annual relapse rate, median (range)	– – –	41 (35–55) 3.5 (1–10) 0.45 (0.05–1.9)	– – –
EDSS score, median (range)	–	4 (2–8)	–
OCBs positive/tested (%) VEP (%) Brain MRI abnormalities (%) Spinal MRI abnormalities (%)	– – – –	4/16 (25) 14/16 (87.5) 6/16 (37.5) 16/16 (100)	– – – –
LESCL (%)	–	16/16 (100)	–

*NMO* neuromyelitis optica, *EDSS* Kurtzke Expanded Disability Status Scale, *OCBs* oligoclonal bands, *VEP* visual evoked potential, *MRI* magnetic resonance imaging, *LESCL* longitudinally extensive spinal cord lesions

### RNA Extraction

For RNA purification, we used TRIzol reagent (Invitrogen, Grand Island, NY, USA) according to the manufacturer’s instructions followed by application of PBMCs to RNeasy spin columns (Qiagen, Venlo, Limburg, Netherlands). Quantification and quality evaluation were performed by using a Nanodrop and Agilent 2100 Bioanalyzer (Agilent Technologies, Santa Clara, CA, USA), respectively.

### lncRNA Microarray

The Human IncRNA Microarray V3.0 (Arraystar, Rockville, MD, USA) was used to design the global profiling of human lncRNAs and protein-coding transcripts. The third-generation lncRNA microarray detects approximately 30,586 lncRNAs and 26,109 coding transcripts. The lncRNAs were carefully constructed using the most highly respected public transcriptome databases (RefSeq, UCSC Known Genes, and Genecode, etc.), as well as landmark publications. A specific exon or splice junction probe was used to represent each transcript that could accurately identify individual transcripts. In addition, positive probes for housekeeping genes and negative probes were printed onto the array to hybridize quality control. GeneSpring GX v11.5.1 software (Agilent Technologies) was used to extract and normalize the data. Volcano Plot filtering and hierarchical clustering were used to identify the differentially expressed lncRNAs and mRNAs that reached the level of statistical significance. Differentially expressed lncRNAs and mRNAs were identified through the random variance model with *P* values calculated by the paired *t* test. The significance cutoff for the upregulated and downregulated lncRNAs and mRNAs was a fold change ≥2.0 with *P* ≤ 0.05.

### qRT-PCR Validation

To validate the microarray data, we randomly selected three upregulated lncRNAs (ENSG00000224298.2, TCONS_00026848, XR_110877.1) from aberrantly expressed lncRNAs. Moreover, we also randomly selected three downregulated lncRNAs (ENST00000563502.1, ENST00000436293.2, and TCONS_00017068).

Quantitative real-time reverse transcription PCR (qRT-PCR) is the gold standard for data verification in this context. For the reverse transcriptase (RT) reaction, SYBR Green RT reagents (Bio-Rad, USA) were used. In brief, the RT reaction was performed for 60 min at 37 °C, followed by 60 min at 42 °C, using oligo (dT) and random hexamers. PCR amplifications were performed by using SYBR Green Universal Master Mix. These reactions were performed in duplicate containing 2× concentrated Universal Master Mix, 1 μL of template cDNA, and 100 nM of primers in a final volume of 12.5 μL, followed by analysis in a 96-well optical reaction plate (Bio-Rad). The lncRNA PCR results were quantified by using the 2^∆∆ct^ method against β-actin for normalization. These data represent the means of three experiments.

We used the following real-time PCR primers: ENSG00000224298.2 CCCAAAGTGCTGGGATTACA; TCONS_00026848 AAACTGAATGGGCAAGGATG; XR_110877.1 CCAGGAGAGGAAGCAGAAGA; ENST00000563502.1 AGGCTCAGGATTTTGCCAGT; ENST00000436293.2 TGTGAATGTGGCTTTGGGTA; TCONS_00017068 CGGTTTGAGTGCTTTTACCAG.

### GO and Pathway Analysis

Gene ontology (GO) analysis was performed to characterize genes and gene products in terms of cellular components, molecular functions, and biological processes. Pathway analysis is an effective method for predicting the underlying biological functions of the differentially expressed genes [10]. This analysis was used to determine the main pathways in which differentially expressed mRNAs underwent significant enrichment. The *P* values and false discovery rate denoted the significance of GO term enrichment and the biological pathways in the differentially expressed mRNA list (recommended *P* value <0.05).

### IncRNA-mRNA Co-Expression Network

The IncRNA-mRNA co-expression network identifies interactions between differentially expressed mRNAs and differentially expressed lncRNAs. The basis of this construct is the normalized signal intensities of specific expression levels of mRNAs and lncRNAs. To formulate the lncRNA-mRNA co-expression network used here, we applied Pearson’s correlations, to calculate statistically significant associations.

### Statistical Analysis

All statistical data were analyzed by using SPSS 17.0 software (SPSS Inc., Chicago, IL, USA). lncRNAs and mRNAs expressed differentially in NMO patients compared to healthy controls were analyzed by using Student’s *t* tests. Statistical significance was considered as *P* < 0.05.

## Results

### lncRNA and mRNA Profiles Differ in Patients with NMO and in Healthy Controls

**Fig. 1:**
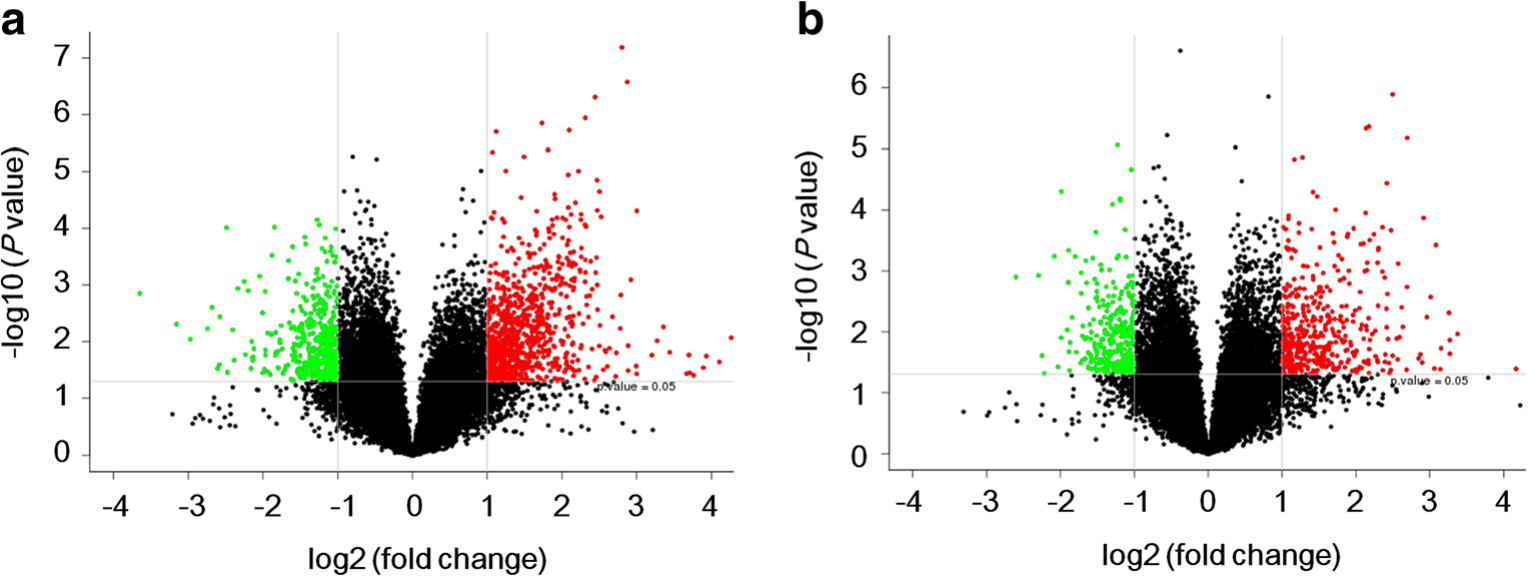
IncRNAs and mRNA profile comparisons between NMO patients and healthy controls. Volcano plots were used to distinguish the differentially expressed lncRNAs (**a**) and mRNAs (**b**). The *vertical lines* correspond to 2-fold upregulation or downregulation, and the *horizontal lines* represent *P* = 0.05. The *red points* highlight the upregulated genes, and the *green points* reflect the downregulated genes

**Fig. 2:**
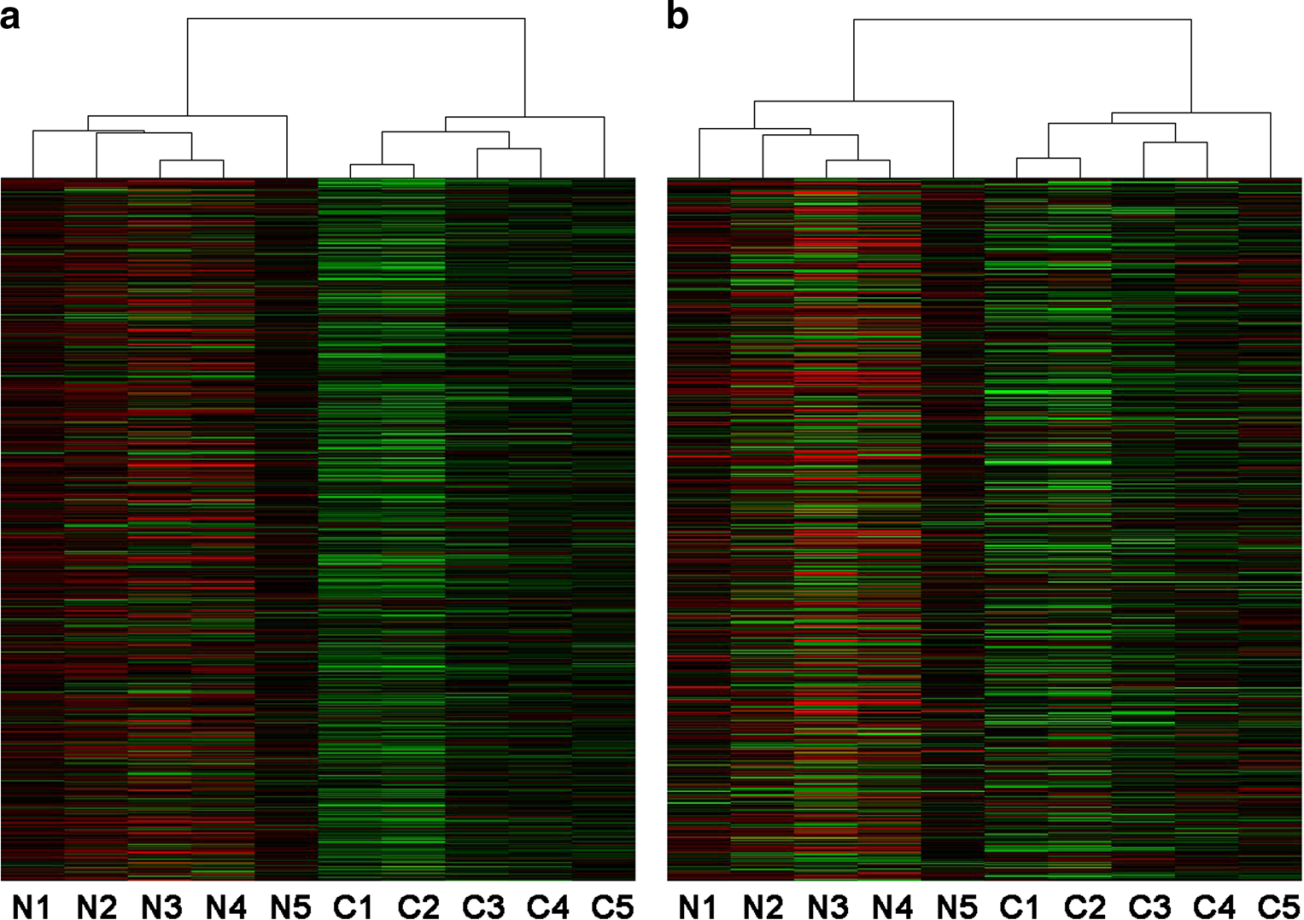
Cluster analysis of differentially expressed lncRNAs and mRNAs of NMO patients and healthy controls. Hierarchical clustering analysis indicated 1310 lncRNAs (**a**) and 743 mRNAs (**b**) that were differentially expressed between NMO patients (N1–N5) and healthy controls (C1–C5). Respectively, the r*ed* and the *green shades* represent the expression levels above and below the relative expression among all samples

Human LncRNA Array v3.0 was used to detect lncRNAs in PBMC from five NMO patients and five healthy controls. Volcano plot analysis was then applied for the direct identification of differences in lncRNAs and mRNAs from these two populations (Fig. 1a, b). Next, a hierarchical clustering technique enabled us to separate the NMO patients from controls in terms of gene expression data (Fig. 2a, b). With a 2/0.5-fold change as the cutoff, a total of 1310 lncRNAs were specifically dysregulated, including 862 upregulated lncRNAs and 448 downregulated lncRNAs, respectively, in NMO patients compared with controls. In addition, 743 differentially expressed mRNAs reached the level of statistical significance in NMO patients compared to healthy controls. Of those, 441 were upregulated and 302 were downregulated.

### qRT-PCR Validation

**Fig. 3:**
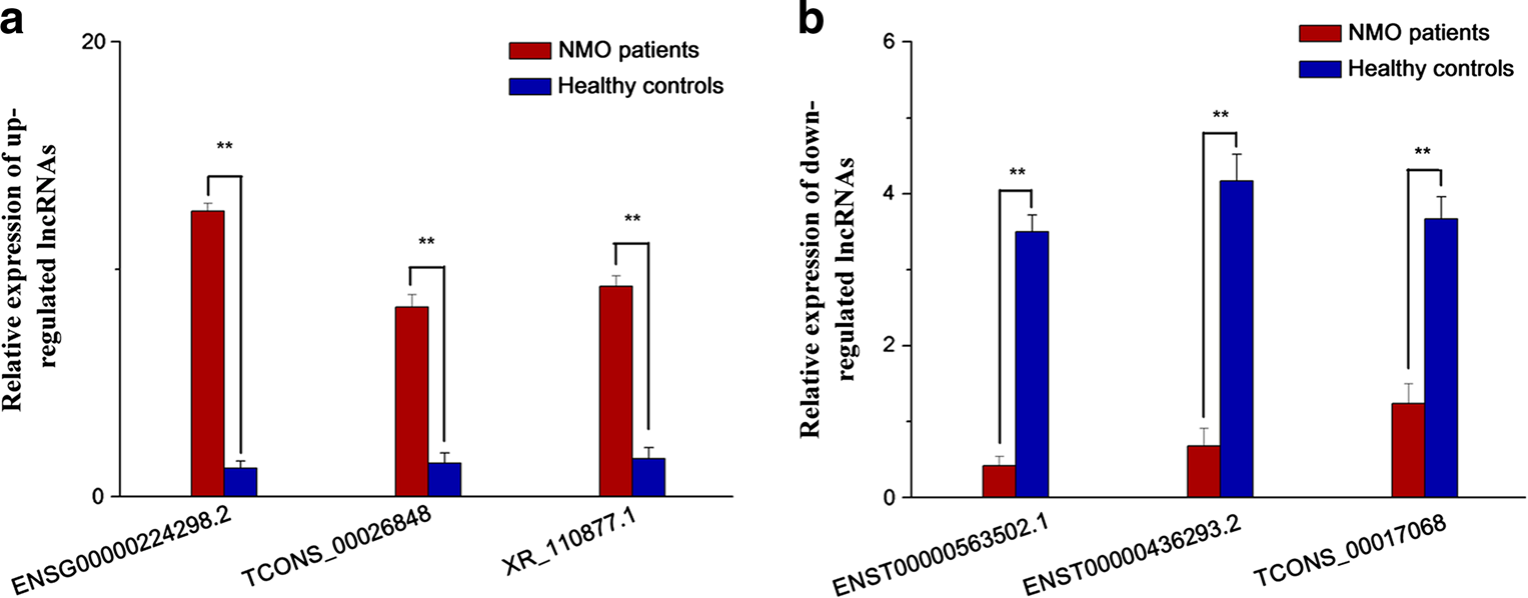
Validation of lncRNA microarray data by qRT-PCR. Three upregulated and three downregulated lncRNAs were validated by qRT-PCR of RNA extracted from PBMCs of 16 NMO patients and 16 healthy controls. The relative expression level of each lncRNA (**a** upregulated lncRNAs, **b** downregulated lncRNAs) was normalized, and data displayed in histograms are expressed as means ± SD, **P < 0.01 comparing NMO patients with healthy controls

To validate our results independently and determine the role of lncRNAs in NMO, we randomly selected six lncRNAs from 862 upregulated lncRNAs and 448 downregulated lncRNAs in NMO patients for comparison to healthy controls. As shown in Fig. 3, of these six lncRNAs that that differed in NMO patients versus healthy controls, IncRNA ENSG00000224298.2 was the most elevated (10.05-fold higher expression), followed by lncRNA TCONS_00026848 (5.68-fold higher expression) and lncRNA XR_110877.1 (5.53-fold higher expression). IncRNAENST00000563502.1, lncRNAENST00000436293.2, and lncRNATCONS_00017068 exhibited 8.3-, 6.13-, and 2.96-fold lower expression, respectively. These results were consistent with those obtained from the microarray chip analyses.

### GO and Pathway Analysis

**Fig. 4:**
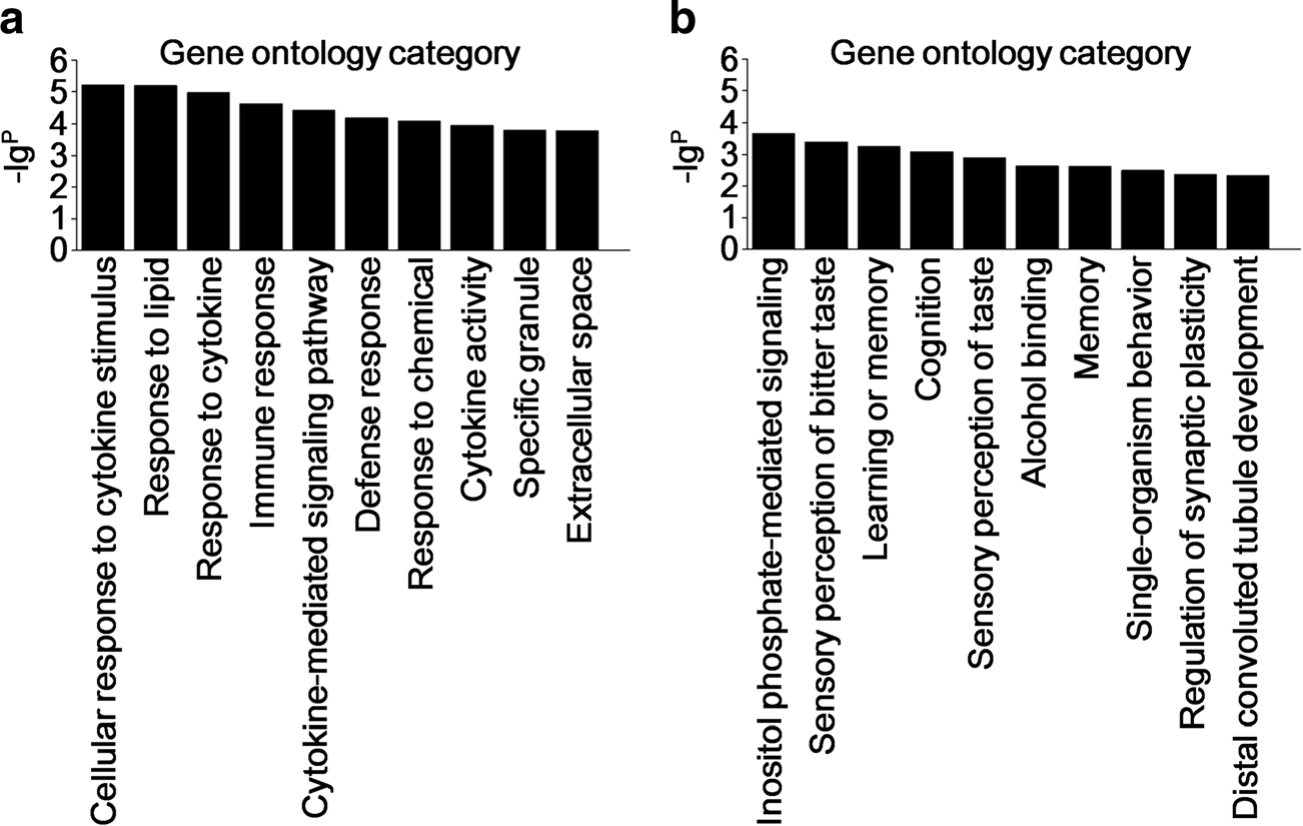
Gene ontology. A total of 743 differentially expressed mRNAs were chosen in GO analysis. The column graphs represent the enrichment of these mRNAs, and the (−lg^P^) value has a positive correlation with GO. **a** The top 10 GOs that were upregulated in the NMO patients compared to the healthy controls. **b** The top 10 GOs that were downregulated in the NMO patients compared to the healthy controls

**Fig. 5:**
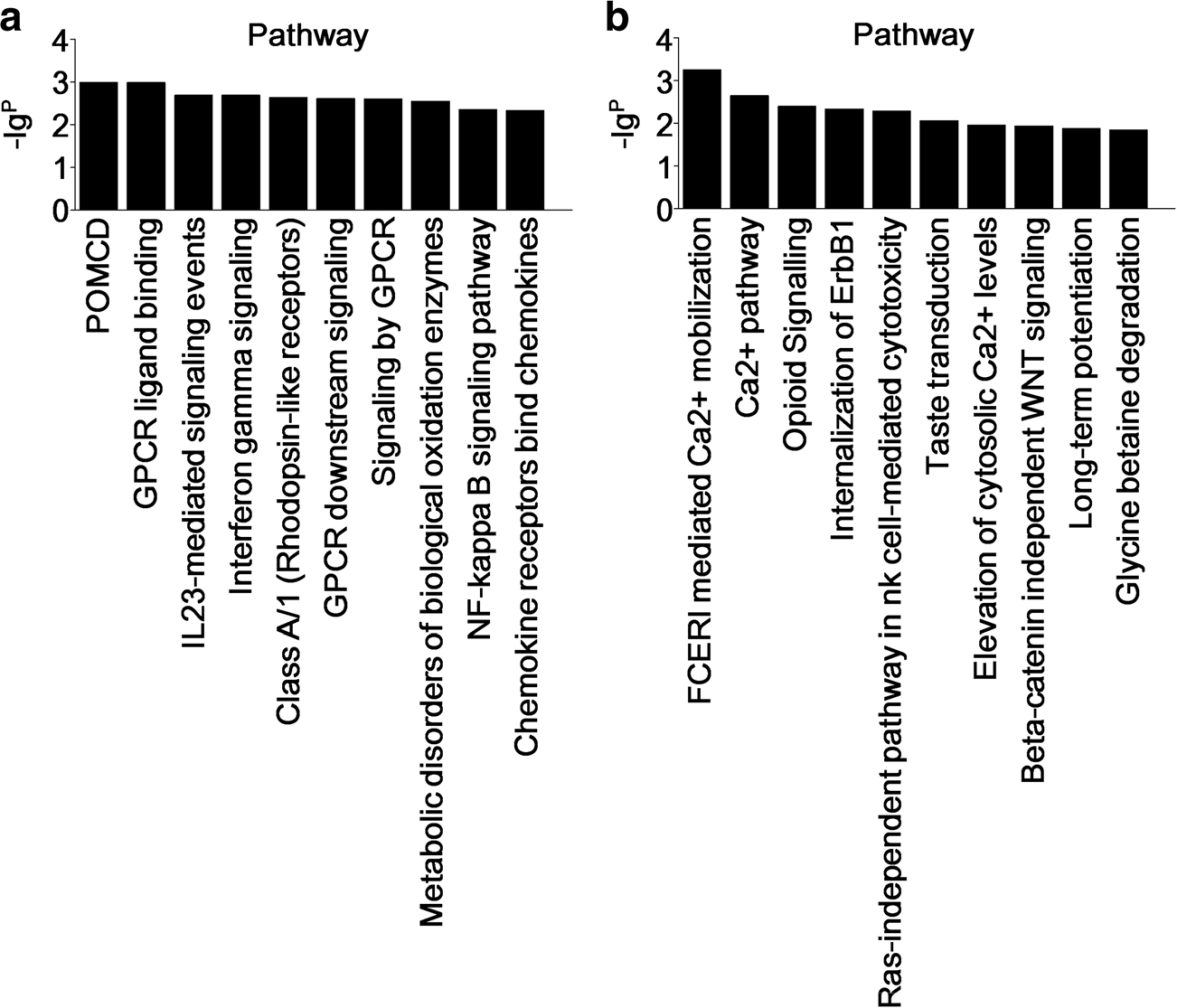
Pathway analyses. A total of 743 differentially expressed mRNAs were chosen in pathway analyses. Column graphs represent the enrichment of these mRNAs, and the (−lg^P^) value has a positive correlation with pathway analyses. **a** The top 10 pathways that were upregulated in the NMO patients compared to healthy controls. **b** The top 10 pathways that were downregulated in the NMO patients compared to healthy controls

To evaluate the enrichment of mRNAs in biological processes, cellular components, and molecular functions, GO analysis was performed on 743 significantly dysregulated mRNAs noted from the microarray outcomes (Supplementary data 1). GO analysis of relevant factors from the upregulated mRNAs included cellular response to cytokine stimulus, response to cytokine, immune response, cytokine-mediated signaling pathway, and response to chemical cytokine activity (Fig. 4a). However, GO analysis of the downregulated mRNAs involved factors of learning or memory, cognition, single-organism behavior, and regulation of synaptic plasticity (Fig. 4b). Subsequent pathway analysis showed that aberrantly upregulated mRNAs were involved in IL23-mediated signaling events, interferon gamma signaling, the natural killer (NK)-κB signaling pathway, chemokine receptors bind chemokines, guanosine-binding protein-coupled receptor (GPCR) ligand binding, and metabolic disorders of biological oxidation enzymes (Fig. 5a). Dissimilarly, results from pathway analysis of the downregulated mRNAs selected long-term potentiation, taste transduction, Ca^2+^ pathway, and the ras-independent pathway in NK cell-mediated cytotoxicity (Fig. 5b) as distinctive features.

### IncRNA-mRNA Co-Expression Network

**Fig. 6:**
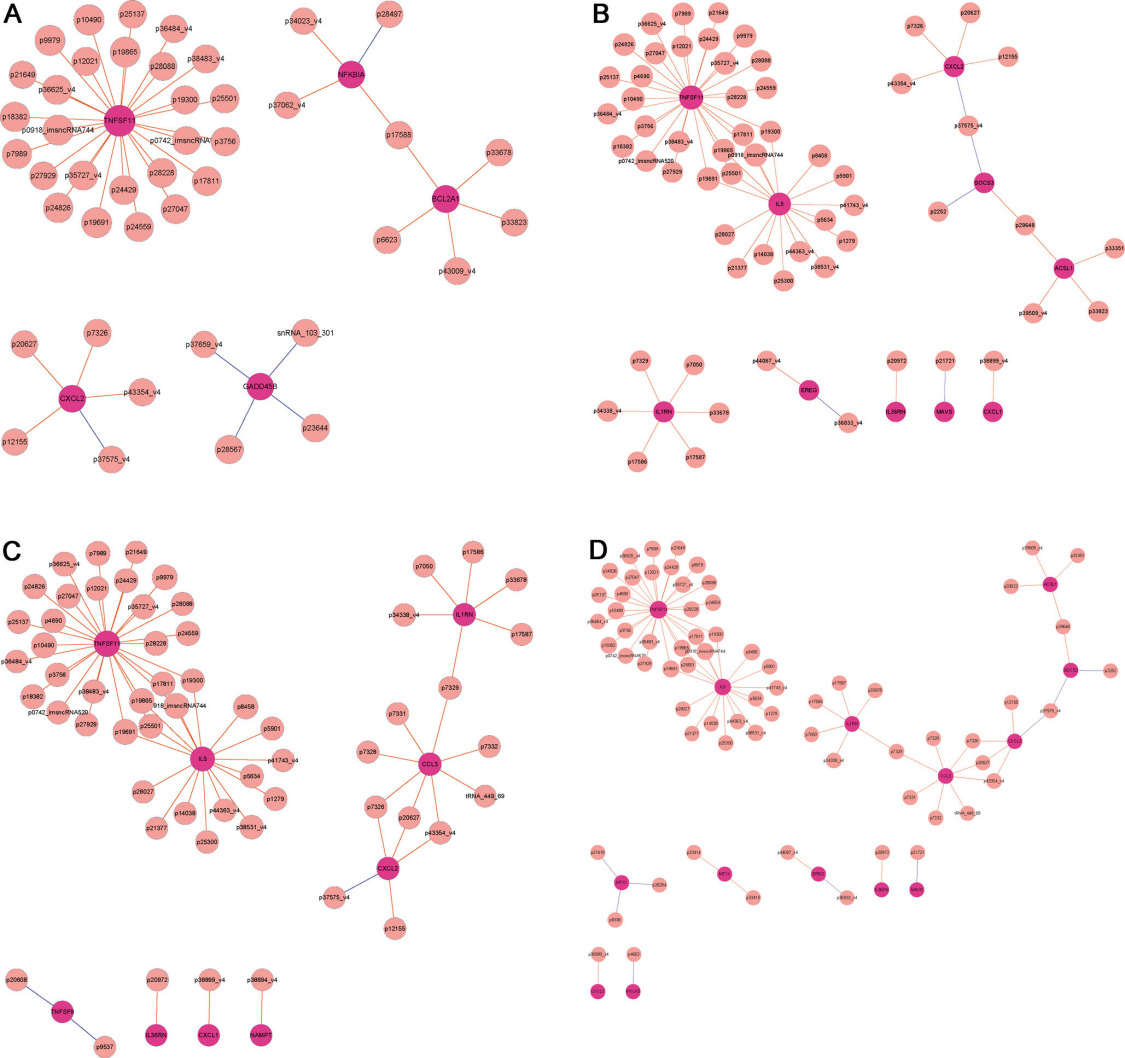
IncRNA-mRNA co-expression network. **a** Forty-three lncRNAs interacted with five mRNAs in the meaningful “NK-κB signaling pathway.” **b** Fifty-nine lncRNAs interacted with ten mRNAs in the GO of “cytokine-mediated signaling pathway.” **c** Fifty-eight lncRNAs interacted with nine mRNAs in the GO of “cytokine activity.” **d** Sixty-nine lncRNAs interacted with 14 mRNAs in the GO of “cellular response to cytokine stimulus”

Co-expression network analysis was then performed to characterize the 1310 differentially expressed lncRNAs and the 743 differentially expressed mRNAs. In total, 183 lncRNAs and 458 mRNAs were included in the co-expression network (Supplementary data 2). Our data showed that the co-expression network was composed of 641 network nodes and 6879 connections. Review of the co-expression network indicated that one mRNA may correlate with 1 to 96 lncRNAs, and one lncRNA may correlate with 1 to 96 mRNAs. Moreover, as Fig. 4 reveals, the lncRNA-mRNA co-expression network was operative in some meaningful GO and pathway analyses. That is, 43 lncRNAs interacted with 5 mRNAs in the “NK-κB signaling pathway” (Fig. 6a), 59 lncRNAs interacted with 10 mRNAs in the GO of “cytokine-mediated signaling pathway” (Fig. 6b), 58 lncRNAs interacted with 9 mRNAs in the GO of “cytokine activity” (Fig. 6c), and 69 lncRNAs interacted with 14 mRNAs in the GO of “cellular response to cytokine stimulus” (Fig. 6d).

## Discussion

With the forefront technology of microarray analysis, we demonstrated here for the first time the expression profiles of human lncRNAs and mRNAs in patients with NMO. Compared to healthy, matched controls, these NMO patients expressed 1310 lncRNAs and 743 mRNAs that were not present in the controls. Moreover, we identified potential functions of these differentially expressed mRNAs with GO and pathway analyses.

IncRNAs are a new class of noncoding RNAs larger than 200 nucleotides. Previously, the involvement of lncRNAs in immune cell development has been reported, including dendritic cell differentiation, T cell activation, granulocytic differentiation, inhibition of T cell proliferation, Th1 cell differentiation, regulation interferon gamma (IFN-γ) expression, regulation of CD4+ Th2 lymphocyte migration, and CD4+ helper T lymphocyte differentiation [7, 11–14]. Moreover, lncRNAs have been recognized as powerful regulators of numerous genes and pathways in the pathogenesis of inflammatory and autoimmune diseases, including SLE, RA, T1DM, MS, autoimmune thyroid disease, psoriasis, and Crohn’s disease [7, 15–17].

NMO is an inflammatory autoimmune disease of the CNS that affects both the spinal cord and optic nerves. Approximately 75 % of NMO patients are seropositive for autoantibodies against the astrocyte water channel aquaporin-4, resulting in astrocyte injury and inflammation [18]. However, because we sought to characterize the epigenetic nature of this disease, the present study was undertaken to investigate the differentially expressed lncRNAs and mRNAs in NMO patients compared to healthy controls.

Designed for the global profiling of human lncRNAs and protein-coding transcripts, the lncRNA microarray V3.0 system was used here to screen the aberrant lncRNAs in five NMO patients to distinguish them from those in five healthy controls. Upon comparing lncRNA and mRNA expression profiles of NMO patients and controls, we found that 1310 lncRNAs (862 upregulated and 448 downregulated lncRNAs) and 743 mRNAs (441 upregulated and 302 downregulated mRNAs) were differentially expressed between NMO patients and healthy controls. In the early stage of NMO, the spinal cord lesion and optic nerve lesion may be not typical, which make it difficult to diagnosis. Therefore, more and more researches focused on finding new technique in early diagnosis of NMO. Our present study may provide basic information of using these differentially expressed lncRNAs and mRNAs in early diagnosis of NMO.

GO and pathway analyses were performed to obtain detailed information on the biological functions and potential mechanisms of these differentially expressed lncRNAs and mRNAs. A total of 743 filtered mRNAs (2-fold change) were included in GO and pathway analyses. GO analysis revealed that 441 upregulated mRNAs were involved in cellular responses to cytokine stimulus, responses to cytokines, immune responses, the cytokine-mediated signaling pathway, responses to chemicals, and cytokine activity (Fig. 4a). However, 302 downregulated mRNAs were participants in learning or memory, cognition, memory, single-organism behavior, and regulation of synaptic plasticity (Fig. 4b). Pathway analysis of the 441 upregulated mRNAs denoted their involvement in IL23-mediated signaling events, IFN-γ signaling, the NF-κB signaling pathway, chemokine receptor binding of chemokines, GPCR ligand binding, and metabolic disorders of biological oxidation enzymes (Fig. 5a). However, 302 downregulated mRNAs were functional in long-term potentiation, taste transduction, the Ca^2+^ pathway, and ras-independent pathway in NK cell-mediated cytotoxicity (Fig. 5b).

Astrocytes, the most abundant cell type in the CNS, are highly sensitive to environmental cues and are implicated in both detrimental and protective outcomes during autoimmune demyelination. As the hallmark cytokine of Th1 cells, IFN-γ plays an important role in the activity of astrocytes in autoimmune inflammation of the CNS. IFN-γ also functioned as a proinflammatory cytokine in the early stage of experimental allergic encephalomyelitis (EAE) [19–21]. Elsewhere, silencing IFN-γ signaling in astrocytes attenuated chemokine expression and inflammatory cell infiltration into the CNS in EAE [22]. In the present study, pathway analysis showed that IFN-γ signaling was upregulated in NMO patients compared with that in healthy controls, which may also indicate an important role of IFN-γ in astrocytes’ functionality of those with NMO. Also, GPCRs have been implicated in elevated astrocyte molecular networks and astrocyte calcium signaling after inflammatory stimuli [23]. In accord, we also found an abundant upregulation of mRNA involvement in GPCR ligand binding. Others have indicated that inhibiting the NF-κB signal transducer pathway can reduce neuroinflammation in astrocytes [24, 25]. In the present study, since the NF-κB signaling pathway was notably upregulated in NMO patients compared with their healthy counterparts, conceivably, the NF-κB signaling pathway could provide a treatment strategy for NMO.

The co-expression network analysis cited here was constructed based on the 1310 differentially expressed lncRNAs and the 743 differentially expressed mRNAs that distinguished NMO patients from control subjects. Our results showed that a total of 183 lncRNAs and 458 mRNAs were included in the co-expression network. Analysis of this co-expression network, which was composed of 641 network nodes and 6879 connections, indicated that one lncRNA could target at most 96 mRNAs, and one mRNA could correlate with at most 96 lncRNAs. We also found that 43 lncRNAs interacted with 5 mRNAs involved in the meaningful NK-κB signaling pathway (Fig. 6a), 59 lncRNAs interacted with 10 mRNAs involved in the GO of cytokine-mediated signaling pathway (Fig. 6b), 58 lncRNAs interacted with 9 mRNAs involved in the GO of cytokine activity (Fig. 6c), and 69 lncRNAs interacted with 14 mRNAs involved in the GO of cellular response to cytokine stimulus (Fig. 6d).

In conclusion, the foregoing outcome, demonstrated here for the first time, suggests that the inter-regulation of lncRNAs and mRNAs may either perpetuate the development of NMO or, alternatively, provide basic information much needed for recognizing and/or alleviating NMO.

## Electronic supplementary material

Below is the link to the electronic supplementary material.

ESM 1 GO and pathway analyses of 441 upregulated mRNAs and 302 downregulated mRNAs. (XLS 614 kb)

ESM 2 Co-expression network analysis of the 1310 differentially expressed lncRNAs and 743 mRNAs. In total, 183 lncRNAs and 458 mRNAs were included in the co-expression network. Moreover, our data showed that the co-expression network was composed of 641 network nodes and 6879 connections. Evaluation of the co-expression network indicated that one mRNA may correlate with 1 to 96 lncRNAs, and one lncRNA may correlate with 1 to 96 mRNAs. (XLSX 944 kb)
